# Chest computed tomography in bronchiolitis obliterans after bone marrow transplantation

**DOI:** 10.1590/0100-3984.2017.50.3e3

**Published:** 2017

**Authors:** Bruno Hochhegger, Matteo Baldisserotto

**Affiliations:** 1 Adjunct Professor of Radiology at the Universidade Federal de Ciências da Saúde de Porto Alegre (UFCSPA), Porto Alegre, RS, Brazil. E-mail: brunohochhegger@gmail.com.; 2 Professor in the Graduate Program of the Faculdade de Medicina da Pontifícia Universidade Católica do Rio Grande do Sul (PUCRS), Porto Alegre, RS, Brasil. E-mail: matteo.baldisserotto@pucrs.br.

Bronchiolitis obliterans (BO) is an inflammatory disease of the small airways, resulting from damage to the lower respiratory tract. The presence of inflammation and fibrosis of the terminal and respiratory bronchioles results in narrowing or complete obliteration of the airway lumen, leading to chronic airflow obstruction^(1,2)^. Histologically, BO is characterized by the presence of intraluminal granulation tissue in the airways or peribronchiolar fibrosis with narrowing of the lumen, provoking a process of scarring and obstruction^(3)^. Although its exact incidence in the pediatric population is unknown, it is known that BO predominantly affects male infants^(4,5)^. Possible causes of BO include inhalation of toxic substances, aspiration syndromes, immunological changes, collagen diseases (rheumatoid arthritis and Sjögren’s syndrome), transplantation, Stevens-Johnson syndrome, and drug reactions. Currently, the diagnosis of BO is based on clinical and computed tomography (CT) criteria, the role of CT being that of excluding the various differential diagnoses^(2)^. BO occurring after bone marrow transplantation (BMT) was first described in 1982^(6)^. It is well known that BO can also occur in lung and heart-lung transplant recipients^(7)^. In BMT recipients, BO appears later than do other pulmonary complications, occurring between 3 and 12 months after transplantation. BO after BMT is more common in patients with chronic graft-versus-host disease, occurring in 6-10% of those who are longterm survivors, with a mortality rate of more than 50%^(8)^.

Chest CT is the most widely used method for the study of interstitial lung diseases and bronchiolar diseases^(9-14)^, having become the tool of first choice because of its great sensitivity and specificity. However, it should be used with great discretion because the patient is exposed to a high dose of ionizing radiation. Diagnostic radiology is considered the main artificial source of radiation to which human beings are exposed, accounting for approximately 14% of the total annual dose received from all sources of radiation^(15)^. Ionizing radiation has the ability to alter the physical and chemical characteristics of the molecules of biological tissues. Cells with a high proliferation rate are more sensitive to ionizing radiation and are found in tissues with high mitotic activity or the so-called fast response tissues. Radiosensitivity is inversely proportional to the degree of cell differentiation (the less differentiated the cell is, the more radiosensitive it is) and directly proportional to the number of cell divisions required for the cell to reach its “mature” stage. In view of these facts, special care should be taken when using CT examinations in children, who are more susceptible to the deleterious effects of radiation than is the rest of the population^(16)^.

In this context, the study conducted by Togni Filho et al.^(17)^, published in the previous issue of **Radiologia Brasileira**, demonstrated that the inspiratory phase can be excluded from the chest CT protocol in children evaluated for post-BMT BO, reducing by half the level of radiation exposure in this population. Their findings are of fundamental importance and have immediate clinical applicability.

## References

[1] Zhang L, Silva FA (2000). Bronchiolitis obliterans in children. J Pediatr (Rio J).

[2] Fischer GB, Sarria EE, Mattiello R (2010). Post infectious bronchiolitis obliterans in
children. Paediatr Respir Rev.

[3] Colom AJ, Teper AM (2009). Postinfectious bronchiolitis obliterans. Arch Argent Pediatr.

[4] Santos RV, Rosário NA, Ried CA (2004). Postinfectious bronchiolitis obliterans: clinical aspects and
complementary tests of 48 children. J Bras Pneumol.

[5] Aguerre V, Castaños C, Pena HG (2010). Postinfectious bronchiolitis obliterans in children: clinical and
pulmonary function findings. Pediatr Pulmonol.

[6] Roca J, Grañeña A, Rodriguez-Rosin R (1982). Fatal airways disease in an adult with chronic graft-versus-host
disease. Thorax.

[7] Paradis I, Yousem S, Griffith B (1993). Airway obstruction and bronchiolitis obliterans after lung
transplantation. Clin Chest Med.

[8] Ralph DD, Springmeyer SC, Sullivan KM (1984). Rapidly progressive air-flow obstruction in marrow transplant
recipients. Possible association between obliterative bronchiolitis and
chronic graft-versus-host disease. Am Rev Respir Dis.

[9] Ribeiro BNF, Ribeiro RN, Zanetti G (2016). Hughes-Stovin syndrome: an unusual cause of pulmonary artery
aneurysms. Radiol Bras..

[10] Mogami R, Goldenberg T, Marca PGC (2016). Pulmonary infection caused by Mycobacterium kansasii: findings on
computed tomography of the chest. Radiol Bras.

[11] Koenigkam-Santos M, Cruvinel DL, Menezes MB (2016). Quantitative computed tomography analysis of the airways in
patients with cystic fibrosis using automated software: correlation with
spirometry in the evaluation of severity. Radiol Bras.

[12] Francisco FAF, Rodrigues RS, Barreto MM (2015). Can chest high-resolution computed tomography findings diagnose
pulmonary alveolar microlithiasis?. Radiol Bras.

[13] Guimaraes MD, Hochhegger B, Koenigkam-Santos M (2015). Magnetic resonance imaging of the chest in the evaluation of
cancer patients: state of the art. Radiol Bras.

[14] Batista MN, Barreto MM, Cavaguti RF (2015). Pulmonary artery sarcoma mimicking chronic pulmonary
thromboembolism. Radiol Bras.

[15] ICRP (1991). 1990 Recommendations of the International Commission on
Radiological Protection. ICRP Publication no. 60. Ann ICRP.

[16] United Nations Scientific Committee on the Effects of Atomic
Radiation (2000). Sources and effects of ionizing radiation. UNSCEAR 2000 Report to the
General Assembly, with Scientific Annexes.

[17] Togni Filho PH, Casagrande JLM, Lederman HM (2017). Utility of the inspiratory phase in high-resolution computed
tomography evaluations of pediatric patients with bronchiolitis obliterans
after allogeneic bone marrow transplant: reducing patient radiation
exposure. Radiol Bras.

